# Assessment of Attractiveness of Cassava as a Roosting Plant for the Melon Fly, *Bactrocera cucurbitae*, and the Oriental Fruit Fly, *B. dorsalis*

**DOI:** 10.1673/031.011.0130

**Published:** 2011-03-18

**Authors:** Grant T. McQuate

**Affiliations:** U.S. Pacific Basin Agricultural Research Center, USDA-ARS, 64 Nowelo Street, Hilo, Hawaii 96720

**Keywords:** bait spray, crop borders, *Manihot esculenta*, *Ricinus communis*, suppression

## Abstract

Application of bait spray to crop borders is a standard approach for suppression of melon fly, *Bactrocera cucurbitae* (Coquillett) (Diptera: Tephritidae) populations and may also be of value for suppression of oriental fruit fly, *B. dorsalis* (Hendel) populations. Establishment of preferred roosting hosts as crop borders may help to improve suppression of both fruit fly species by providing sites for bait spray applications. In an area-wide *B. cucurbitae* suppression trial, the question was raised as to whether cassava, *Manihot esculenta* Crantz (Euphorbiales: Euphorbiaceae), could be used as a *B. cucurbitae* roosting host. *M. esculenta* was of interest as a roosting host because, in contrast to many other identified preferred roosting hosts, it would also be a crop potentially increasing the productivity of the crop production system overall. As a short-lived and shrubby perennial, *M. esculenta* potentially constitutes a crop with more persistent roosting foliage than an annual crop such as corn, *Zea mays* L. (Cyperales: Poaceae), that has often been planted as a roosting host for *B. cucurbitae* control. Using protein-baited traps set amidst potted plants placed adjacent to a papaya *Carica papaya* L. (Violales: Caricaceae) orchard known to have established populations of *B. cucurbitae* and *B. dorsalis*, the effectiveness of *M. esculenta* as a roosting host was assessed by comparing its attractiveness to that of castor bean, *Ricinus communis* L (Malpighiales: Euphorbiaceae), previously identified as one of the most attractive roosting hosts for *B. cucurbitae*, and to corn, a crop which has been planted as a roosting host for help in *B. cucurbitae* control. The results showed that use of *M. esculenta* as a roosting host is comparable to use of *R. communis* by both *B. cucurbitae* and *B. dorsalis.* These results provide encouragement to incorporate *M. esculenta* on a farm as a trap crop (i.e. site for bait spray application). This has the advantage of having the trap crop be a crop on its own (as opposed to castor bean) and, among prospective crops that could be used as a trap crop, has foliage more persistent than an annual trap crop such as corn.

## Introduction

Protein bait sprays that incorporate a toxicant are commonly used for suppression of tephritid fruit flies. For control of melon fly (*Bactrocera cucurbitae* (Coquillett) (Diptera: Tephritidae)) populations, bait sprays are typically applied to vegetation bordering agricultural host areas where the adults seek shelter (“roost”). Application of bait spray to crop borders may also be of value for suppression of oriental fruit fly, *B. dorsalis* (Hendel) populations, especially in relation to certain hosts such as papaya, *Carica papaya* L. (Violales: Caricaceae) (Stark 1995; McQuate and Vargas 2007). A number of plants have previously been identified as preferred roosting hosts of these two tephritid fruit fly species (Nishida and Bess 1957; Kazi 1976; Stark 1995; McQuate *et al.* 2003; McQuate and Vargas 2007). Establishment of preferred roosting hosts as crop borders may help to improve suppression of both fruit fly species by providing sites for bait spray applications. If no good roosting hosts are available near a host crop it is difficult to control the fly populations through the use of bait sprays because one does not know where the flies go for food and shelter. If, however, bait sprays are not used for population control, establishment of preferred roosting hosts could conceivably aggravate problems with Tephritid fruit flies by producing a more favorable environment for the flies.

Following the completion of an earlier comparative assessment of potential roosting hosts (McQuate and Vargas 2007), questions were raised about the potential value of cassava, *Manihot esculenta* Crantz (Euphorbiales: Euphorbiaceae), as a roosting host for *B. cucurbitae. M. esculenta* had not been one of the plant species included in the earlier trials. This question was raised in Mauritius over the course of an International Atomic Energy Agency-supported technical cooperation field project (“Feasibility Study for the Suppression of the *B. cucurbitae* in Selected Areas of Mauritius”). *M. esculenta* was of interest as a roosting host because, in contrast to many other identified preferred roosting hosts, it would also be a crop, potentially increasing the productivity of the crop production system overall. As a short-lived, shrubby perennial (Tindall 1983), *M. esculenta* potentially constitutes a crop with more persistent roosting foliage than an annual crop such as corn, Zea mays L. (Cyperales: Poaceae), which has often been planted as a roosting host for *B. cucurbitae* control (Nishida and Bess 1957; Kazi 1976). In the course of an area-wide *B. cucurbitae* suppression trial on the island of Oahu (Hawaii, USA), it was noted that melon flies did roost in *M. esculenta* foliage, but seemed to prefer it less than *Z. mays* foliage (Ron F.L. Mau, University of Hawaii, personal communication). Here, research is reported which is designed to assess the effectiveness of *M. esculenta* as a roosting host. The attractiveness of *M. esculenta* as a roosting host is compared to that of castor bean, *Ricinus communis* L (Euphorbiales: Euphorbiaceae), identified as one of the most attractive roosting hosts for *B. cucurbitae* (McQuate and Vargas 2007), and to *Z. mays*, a crop which has been planted as a roosting host for help in *B. cucurbitae* control. Because the site selected for the study (see below) had a well-established *B. dorsalis* population and a well-established *B. cucurbitae* population, it was possible to assess the use of vegetation in crop borders by both *B. cucurbitae* and *B. dorsalis.*

## Materials and Methods

**Figure 1.**
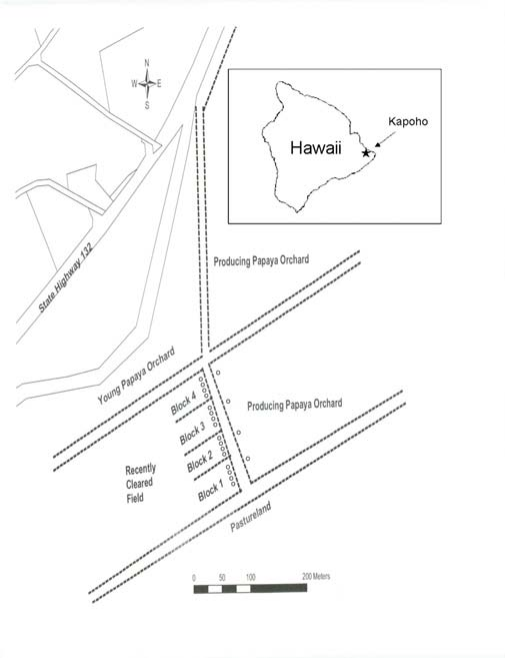
Locations of fields in which potted plants were placed relative to *C. papaya* orchards. Circles indicate locations of protein baited traps. High quality figures are available online.

### Study Site

Field trials were conducted in a fallow field adjacent to a *C. papaya* orchard in Kapoho, Hawaii (see Figure 1). *C. papaya*, a tropical crop, is produced year-round in orchards at this site. The orchard adjacent to the trial was producing ripe fruits and had varying levels of ground fruits (i.e. ripe fruits that had fallen to the ground) that supported large wild populations of both *B. cucurbitae* and *B. dorsalis* (Liquido 1991, 1993).

### Plant Species Tested

*M. esculenta* plants were grown from cuttings of an unnamed cultivar found in Hawaii. At the time of the field trial, plants (maintained as one plant per 26 liter pot) averaged 1.18 ± 0.03 m high. *R. communis* plants were grown from seed collected from plants in the vicinity of Laie on the island of Oahu. The plants (also planted one plant per 26 liter pot) averaged 1.16 ± 0.04 m high. The *Z. mays* variety, Hawaiian Supersweet #9, was planted from seed purchased locally and was thinned to three plants per 26 liter pots. Plants averaged 0.74 ± 0.02 m high at the time of the trial.

**Figure 2.**
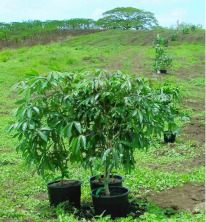
Overview of trial set-up with *C. papaya* orchard to the left, and protein baited traps - placed both with and without association to clusters of pots of test plants arranged 20 m from the border of the papaya orchard. High quality figures are available online.

### Bioassay

**Figure 3.**
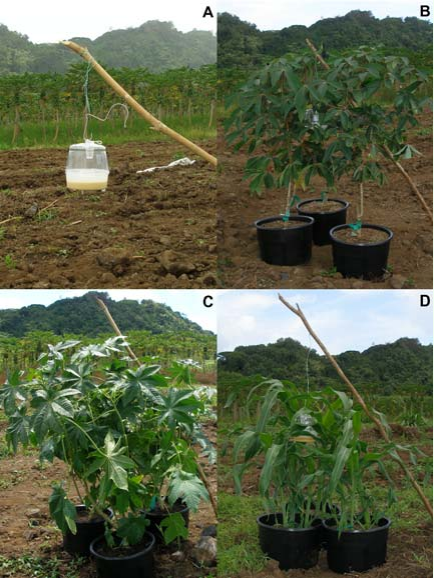
(A) Control protein bait trap (placed without association to potted plants) in open field 20 m from the border of the *C. papaya* orchard; (B) Close-up of *M. esculenta* cluster showing central positioning of protein bait trap; (C) Close-up of *R. communis* cluster showing central positioning of protein bait trap; and (D) Close-up of *Z. mays* cluster showing central positioning of protein bait trap. High quality figures are available online.

On 4 June 2008, plants of each species tested were set out in a fallow field along a line 20 m from the edge of an adjacent *C. papaya* orchard (see Figure 2). The distance chosen from the *C. papaya* orchard had been found to yield fly response to the plant cluster, but limited direct response to the protein bait trap (McQuate and Vargas 2007). A clear bottom Multilure trap (Better World Manufacturing, www.abettertrap.com) baited with a protein bait solution [8% Solulys (Roquette America, Inc., www.roquette.com); 4% Borax; 88% water] was hung within each cluster of plants. In addition to the plant clusters, four similarly treated protein bait traps were hung without association to any plants (blank). The latter traps provided a control for attraction to the bait only. Protein-baited traps were chosen for the assessment of fly presence over direct observations of flies in the foliage both because fly numbers at any given time may be low (and the traps provided a means of accumulating numbers over time) and because of difficulties of reliably detecting all of the flies present throughout the foliage in the plant cluster. Furthermore, the traps incorporated a two step process of fly response to foliage followed by fly response to bait as must happen in order for bait sprays to be effective in population suppression. Figure 3A shows a “blank trap” (trap hung without association to any plants); a cluster of cassava plants with an associated trap (Figure 3B); a cluster of castor bean plants with an associated trap (Figure 3C); and a cluster of corn plants with an associated trap (Figure 3D). Plant clusters (three pots for each plant species) and blank traps, were set in 4 blocks, each block with four stations, one for each treatment. Stations were placed 8m apart within the row (see Figure 1). Position within each block was determined randomly. Protein bait traps (4) were also placed between the second and third tree in from the edge of the *C. papaya* orchard to monitor the source tephritid fruit fly population levels. All protein bait traps were serviced every 2 days with location of all plant clusters and protein bait only traps in the fallow field moved to a new ‘random’ orientation every 4 days within each block (traps in the *C. papaya* orchard were not moved). A total of 4 rotated positions was completed, giving a total of 5 trap servicing cycles overall, with the last trap service on 24 June 2008. Over the course of the position rotations, each treatment was positioned at each site of the block at least one time. Protein bait traps were “topped-off” with fresh protein bait solution at each service and totally replaced after 12 days (3, 4-day cycles).

### Calculation of Leaf Areas of Test Plant Species

In order to permit standardization of catch by leaf area (because equivalent leaf areas could not readily be presented for all species tested), total leaf area was estimated for each plant cluster but the technique used differed among plant species depending on the size and shape of the leaves. For *Z. mays*, leaf area was estimated using a CI-203 portable laser area meter (CID Bio-Science Inc., www.cidinc.com). Shape and size of *M. esculenta* and *R. communis* leaves made it difficult to use the leaf area meter directly for leaf area measurements. Consequently, for these plants, leaf area was approximated using a method similar to a leaf area estimation procedure used with *Z. mays* (Zhang and Brandle 1997) where area was estimated by the sum of the products from each leaf of leaf length (*L*), maximum leaf width (*W*), and a correction factor. For *M. esculenta* and *R. communis*, the following regressions were used to estimate leaf area:

*M. esculenta* leaf area (cm^2^) = 0.0731 × (no. of lobes) × (leaf central lobe length [cm])^2^ + 47.716

*R. communis* leaf area (cm^2^) = 23.135 × (maximum leaf width) - 248.847

Leaf areas for these regressions were obtained by cutting leaves into pieces that were small enough (maximum dimension < 15.0 cm) to be measured by the CI-203 portable laser area meter, set up with a CI-203CA conveyor attachment (CID Bio-Science). These regressions had r^2^ values of 0.81 (*M. esculenta*) and 0.96 (*R. communis*).

### Statistical Analyses

The two 2-day trap catches for each protein bait trap (both those associated with plants and those not associated with plants) were combined for each of the five cycles, effectively providing an average catch response for each cycle. Catch was converted to flies per trap per day before data transformation and analysis. Average trap catch for each cycle was square root transformed [√ (x + 0.5)] (Sokal and Rohlf 1981) and subjected to analysis of variance (ANOVA), with the Tukey-Kramer HSD Test for means separation (JMP 2007). Square root transformed catch per trap per day divided by leaf area was also analyzed by ANOVA, with Tukey-Kramer HSD for means separation (JMP 2007). Percentage female catch from each cycle was arcsine transformed [arcsin (√ (%/100))] (Sokal and Rohlf 1981) and subjected to analysis of variance (ANOVA) (JMP 2007). Figures summarizing bioassay results present untransformed trap catch results together with statistical results based on transformed values.

**Figure 4.**
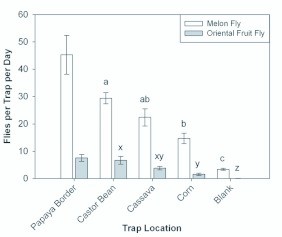
Average catch of *B. cucurbitae* and *B. dorsalis* per trap per day in protein-baited traps placed in the *C. papaya* orchard; and in clusters of *R. communis* plants, *M. esculenta* plants, and *Z. mays* plants; and without association to plants (blank) 20 m away from the *C. papaya* orchard. Columns of the same shading which have the same letter are not significantly different at the α = 0.05 level. Average trap catch in the *C. papaya* orchard is presented as an indication of the size of the source population, but was not included in the ANOVA because traps were not regularly repositioned as done for traps associated with the three plant species and the traps without association with plants (blank traps). High quality figures are available online.

## Results

**Figure 5.**
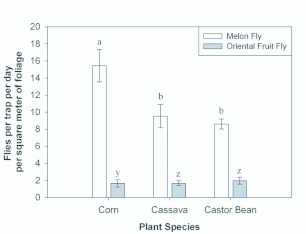
Average catch of *B. cucurbitae* and *B. dorsalis* per trap per day per square meter of *Z. mays* foliage, *M. esculenta* foliage, and *R. communis* foliage. Columns of the same shading which have the same letter are not significantly different at the α = 0.05 level. High quality figures are available online.

There were significant differences in trap catch among treatments for both *B. cucurbitae* (*F* = 39.65; df = 3, 16; *p* < 0.0001) and *B. dorsalis* (*F* = 24.64; df = 3,16; *p* < 0.0001). The trend in catch among treatments was the same for both species: catch dropping off through *R. communis, M. esculenta, Z. mays*, and blank treatments. Significance of difference in catch among treatments was also the same between species. For both species, catch in *R. communis* was higher, but not significantly different from catch in *M. esculenta* and catch in *M. esculenta* was higher, but not significantly different from catch in *Z. mays.* However, catch in *R. communis* was significantly higher than catch in *Z. mays*, and catch in any of the three plant species was significantly greater than the catch in the blank. For both fly species, catch in the *C. papaya* orchard was greater than catch associated with any of the plant species tested. However, because positions of the traps in the *C. papaya* orchard were not rotated as with the three plant species and the blanks, the trap catch was not included in the ANOVA, so no test was made to assess significance of difference of catch in the *C. papaya* orchard relative to catches associated with the plant species. Average trap catch results, together with ANOVA results, are presented in Figure 4. The average fly catch per square meter of foliage was also significantly different for both *B. cucurbitae* (*F* = 59.23; df = 2, 12; *p* < 0.0001) and *B. dorsalis* (*F* = 17.72; df = 2,12; *p* = 0.0003). For both fly species, catch was significantly higher for the leaf area adjusted corn foliage than for either area-adjusted *M. esculenta* foliage or area-adjusted *R. communis* foliage. There was no significant difference in leaf area — adjusted catch between *M. esculenta* and *R. communis* plant clusters (see Figure 5). For *B. cucurbitae*, the proportion of female catch was significantly different among plant species (*F* = 4.49; df = 3, 76; *p* = 0.0059). It was greatest for *Z. mays*, but differences were not significant among *Z. mays, C. papaya*, and *M. esculenata.* Though, the proportion of female catch was significantly less for *R. communis* than for either *Z. mays* or *C. papaya.* For *B. dorsalis*, the proportion of female catch was also significantly different among plant species (*F* = 3.37; df = 3, 74; *p* = 0.023). It was greatest for *M. esculenta*, but differences were not significant among *M. esculenta, Z. mays*, and *C. papaya.* The proportion of female catch was, though, significantly less for *R. communis* than for *M. esculenta.*

## Discussion

This study has shown that use of *M. esculenta* as a roosting host is comparable to use of *R. communis* by both *B. cucurbitae* and *B. dorsalis.* Although there was higher catch associated with *R. communis* for both species, the catch difference was not significant. These results provide encouragement to incorporate *M. esculenta* on a farm as a trap crop (i.e. site for bait spray application). This has the advantage of having the trap crop be a crop on its own (as opposed to *R. communis*) and, among prospective crops that could be used as a trap crop, has foliage more persistent than a trap crop that is an annual crop such as *Z. mays.*

Protein baited traps placed in *Z. mays*, which had previously been identified as an attractive plant for melon flies (Nishida and Bess 1957; McQuate *et al.* 2003), had lower catch for both fruit fly species than traps placed in either *R. communis* or *M. esculenta* (though the difference with *M. esculenta* was not significant). However, after trap catch was adjusted for leaf area, trap catch was significantly higher in *Z. mays* than in either *M. esculenta* or *R. communis.* These results are suggestive that larger clusters of *Z. mays* could have improved attractiveness relative to *R. communis* and *M. esculenta*, especially considering that the average leaf area (± SEM) for *Z. mays* in this study (0.94 ± 0.048) was significantly less than the average leaf area for *M. esculenta* (2.54 ± 0.28) which was significantly less than the average leaf area for *R. communis* (3.45 ± 0.12) (*F* = 51.13; df = 2,9; *p*<0.0001). The importance of increased breadth of a roosting host was earlier reported by Prokopy *et al.* (2004) where it was noted that bait spray application to narrow sorghum borders was less effective for *B. cucurbitae* control than application to broader sorghum borders. Considering the results for *Z. mays*, it is interesting to note that the leaf area adjusted catch for *M. esculenta* was not significantly different from the leaf area adjusted catch for *R. communis*, even though the average leaf area for *M. esculenta* was significantly less than the average leaf area for *R. communis*.

Application of a bait spray on *M. esculenta* foliage would not be expected to affect the marketability of the root, especially if an environmentally friendly bait spray such as the spinosad-based GF-120 NF Fruit Fly Bait (Dow AgroSciences, Indianapolis, IN) is used, which has passed review by The Organic Materials Review Institute (OMRI) for use in organic production. There may, however, be some concern if the *M. esculenta* leaves are also harvested for consumption, as is common in Zaire, Indonesia, Malaysia, and parts of South America (e.g. Brazil). *M. esculenta* as a leaf vegetable is, however, more commonly grown for home production than for marketing (Oomen and Grubben 1978).

Some plant species used as roosting hosts by *B. cucurbitae* and *B. dorsalis* (e.g. castor bean and wiliwili [*Erythrina variegata* L. (Fabales: Fabaceae)]) have been observed to have extra-floral nectaries (McQuate and Vargas 2007) and *B. cucurbitae* and *B. dorsalis* have been observed to feed on nectar from extra-floral nectaries in *R. communis* (Nishida 1958). *M. esculenta* is another plant species that may have extra-floral nectaries. Ogburia (2004) found that five *M. esculenta* clones established in a greenhouse possessed functional extra-floral nectaries in petioles, leaves, stipules, and stems; and the extrafloral nectaries had nectar exudates. However, these same clones established in the field possessed non-functional extra-floral nectaries that had no nectar exudates. Gary and Foster (2004) indicated that *M. esculenta* “can produce copious amounts of extra-floral nectar at the leaflet tips” and Pereira and Splittstoesser (1987) observed that “the presence of small sugary translucid droplets, hanging from the base of the petiole and abaxial side of the veins of cassava leaves, is a common phenomenon seen during early morning in plants growing in the field or glasshouse.” Although extra-floral nectaries were not observed in the present trial, they may be present on certain *M. esculenta* varieties and/or be functional under certain environmental conditions such as conditions of higher humidity as may be found in a greenhouse environment. Nectar exudates from extra-floral nectaries may increase the value of *M. esculenta* as a roosting host by providing a food source.

One issue not addressed in this study is whether differences in stage of plant phenology would affect the relative attractiveness of these three plant species. It has been noted that both *B. cucurbitae* and *B. dorsalis* may show increased population levels in *Z. mays* at the time of, and subsequent to, flowering and pollen shed (McQuate et al. 2003). All plants in this study, though, were maintained in a non-flowering stage.

As noted in a previous publication on border plant use by *B. cucurbitae* and *B. dorsalis* (McQuate and Vargas 2007), identification of attractive non-hosts provides a basis for one means of improving the effectiveness of bait sprays for *B. cucurbitae* control suggested by Prokopy et al. (2003). This issue is also true for *B. dorsalis*, if not also for other tephritid fruit fly species as well. Because the sexual maturity or protein status of the attracted flies was not determined, it is not known whether these plants are attractive to both proteinsatiated and protein-hungry females and the favored plant choice for improved effectiveness of bait sprays (Prokopy et al. 2003).

Further research is needed to document the relative attractiveness of different stages of phenology of plant species used as roosting sites as well as the effect of sexual maturity or protein status on the use of different roosting hosts. Additionally, further research is needed on the effectiveness of different border densities for different roosting hosts as well as differences in planting pattern (e.g. continuous rows versus patches and distance between adjacent patches). As understanding of roosting behavior improves, it will be easier to establish priorities for species selection for crop borders as well as to improve the overall targeting of bait sprays to optimize population suppression of these tephritid fruit fly species.
